# Bidirectional Relationship Between Dental Diseases and Sleep Disturbances in Pediatric Patients

**DOI:** 10.1002/cre2.70191

**Published:** 2025-08-12

**Authors:** Yaqiong Zhang, Wenqi Yang, Qi Sun, Fangkai Han, Minjun Dong

**Affiliations:** ^1^ Department of Oral and Maxillofacial Radiology, Shanghai Stomatological Hospital & School of Stomatology Fudan University Shanghai China; ^2^ Department of Radiology, School of Medicine, Ninth People's Hospital Shanghai Jiao Tong University School of Medicine Shanghai China

**Keywords:** dental disease, obstructive sleep apnea, oral hygiene, pediatric patients, sleep disturbance

## Abstract

**Background:**

Certain dental diseases in pediatric patients may disturb their sleep, affect their oral health‐related quality of life, and result in a negative influence on cognition and behavior. On the other hand, sleep disturbances may also increase the risk or participate in development and progression of dental diseases.

**Objective:**

This narrative review aimed to overview of the bidirectional relationship between common dental diseases and sleep disturbances, as well as the potential mechanisms behind.

**Material and Methods:**

PubMed, Web of Science, and Google Scholar were searched using the keywords “dental disease,” “sleep disturbances,” and “children,” and only articles published in English were included.

**Results:**

Evidence provided by previous studies has indicated that common dental diseases, including dental caries, temporomandibular disorders, and dentofacial deformities, induced sleep disturbances in children and adolescents. On the other hand, common sleep disturbances such as sleep disordered breathing, obstructive sleep apnea, as well as other sleep problems, including sleep bruxism and sleep profile impairments, have a strong link to oral health conditions in pediatric patients. Alteration of oral microorganism colonization, impairment in the immune system, persistent inflammation, and chronic pain have contributed to sleep disorders triggered by these dental diseases.

**Conclusion:**

Upon identification of dental caries, gingivitis, periodontitis, as well as other dental problems, a checkup on a child's sleep is important, as this may subsequently affect his/her initiation, maintenance, duration, and quality of sleep. Dentists and orthodontists could play a critical role in early detection, prevention, and intervention of the dental health‐related sleep disturbances.

**Abbreviations**
AHI: apnea hypopnea index
BOP: bleeding on probing
COHIP: child oral health impact profile
DMF: decay‐missing‐filled
FAIREST‐6: functional airway evaluation screening tool
OHRQoL: oral health‐related quality of life
OSA: obstructive sleep apnea
PPD: pocket probing depth
PSQ: Pediatric Sleep Questionnaire
RME: rapid maxillary expansion
SAHS: sleep apnea‐hypopnea syndrome
SDB: sleep disordered breathing
SDIS: Sleep Disorders Inventory for School
SDSC: Sleep Disturbance Scale for Children
TMD: temporomandibular disorder
TMJ: temporomandibular joint

## 1 Introduction

The prevalence of dental diseases such as dental caries and dentofacial deformities is high in children. These diseases not only affect a child's quality of life but also cause disturbance of sleep. Previous studies have revealed a considerable proportion of children suffering from sleep disturbances due to dental problems (Lima et al. 2018; Moro et al. 2020). For instance, dental caries, temporomandibular disorders (TMD), mouth breathing, and dentofacial deformities have all been previously shown to increase the risks or represent features of certain sleep disorders (Ogawa et al. 2021; Palermo et al. 2011; Ikavalko et al. 2018; Flores‐Mir et al. 2013), which may subsequently affect the cognitive and psychological development of children (Suratt et al. 2007). On the other hand, sleep disturbances may also increase the risk or participate in development and progression of dental diseases (Chen et al. 2018). Therefore, the correlations between dental diseases and sleep disturbances may be bidirectional. Except for the well‐acknowledged correlation between dentofacial deformities and sleep disordered breathing (SDB), the correlations between other dental diseases and sleep disturbances remain unclear.

Several hypotheses have been proposed to link dental diseases and sleep disturbances mechanistically. In addition to the contribution of adenoid hypertrophy, children with abnormal dentofacial structures such as mandibular retrusion with posterior rotation increase the risk for SDB (Flores‐Mir et al. 2013), indicating that a change in morphology may contribute to the development of SDB. However, the mechanisms underlying the bidirectional correlation between dental diseases and sleep disturbances remain to be further elucidated.

Given that children and adolescents are at a specific stage in life, early identification and intervention of these diseases may potentially bring benefits both in the short term and in the long term. For instance, the persistent snoring of young patients with obstructive sleep apnea (OSA) has been revealed to correlate with hyperactivity, inattention, and poorer cognitive development (Beebe et al. 2012), and subsequent problems in behavior, emotion, and sustained attention (Beebe 2006). SDB may also manifest as inattention and hyperactivity during daytime, while sleep deprivation in children may manifest as a change in the mood and temperament (Gruber et al. 2014; El‐Sheikh et al. 2007; Gregory and Sadeh 2012; Waxmonsky et al. 2017). Previous studies have already shown that treatment initiated at as early as 6 months of age will improve behavior significantly in later life (Bonuck et al. 2012). Features flagging for potential risk of SDB in children could be screened by the Functional Airway Evaluation Screening Tool (FAIREST‐6) (Heit et al. 2022), whereas the Pediatric Sleep Questionnaire (PSQ) and Sleep Disorders Inventory for School (SDIS) could be utilized to assess risk of OSA in children (Chervin et al. 2000).

Dentists are in a unique position to identify the potential risks as well as symptoms of sleep disturbances through dental visits. Therefore, a thorough understanding of the prevalence, risks, correlations, and mechanisms between dental diseases and sleep disturbances in children is crucial to facilitate disease prevention and intervention. This study will provide a critical review of the current research status on the correlations between dental diseases and sleep disturbances, and the research progress in the identification of underlying mechanisms for these correlations.

## 2 Methods

PubMed, Web of Science, and Google Scholar were searched using the keywords “dental disease,” “sleep disturbances,” and “children,” and only articles for humans and published in English were included for further relevance screening. No time restrictions were applied. Records were screened by two independent researchers, whereas a third reviewer was consulted when discrepancies were present, and a final agreement was achieved after discussion.

## 3 Dental Diseases Induce Sleep Disturbances

Previous studies have revealed a close relationship between dental diseases and sleep disturbances. Approximately 6.6% of children aged 2–5 years with certain dental problems are affected by sleep disturbances (Gomes et al. 2014). If oral conditions (such as dental caries) are left untreated, 72.8% of these children will undergo trouble sleeping (Lima et al. 2018). Among children aged 8–10 years, 28% cases with sleep disturbances are caused or at least partially caused by dental issues, including dental caries, pulpal involvement, ulceration of tissue due to tooth fragments from decayed crowns, fistula, abscesses, and traumatic dental injuries (Moro et al. 2020). The prevalence of sleep problems is even higher in adolescents with oral health conditions, reaching around 33% of adolescents at 12 years of age (Souza et al. 2018). In addition, worsening of oral hygiene induced higher scores in the Sleep Disturbance Scale for Children (SDSC), especially for domains of sleep breathing disorders, sleep‐wake transition disorders, and disorders of excessive somnolence (Baptista et al. 2021). Based on the above evidence, dentists and orthodontists are in a crucial position to identify sleep disturbances. A previous study has revealed that children with an average age of 9.2 years present a higher risk of SDB when having comorbidity of respiratory disorders and orofacial symptoms (Baidas et al. 2019), and multicenter study has demonstrated that among pediatric patients aged 5–17 years seeking orthodontic treatment, 29.5% were at high risk of OSA (Choong et al. 2023).

### 3.1 Dental Caries

Dental caries is one of the most common dental diseases in children, and if left untreated in either deciduous or permanent teeth, it often leads to pain, discomfort, and infection, which subsequently affects initiation and maintenance of sleep in children (Gomes et al. 2014; Souza et al. 2018). A multicenter clinical study has revealed that in children at 3–6 years of age, the number of teeth with caries negatively affects sleep duration (Ogawa et al. 2021), and if left untreated in either deciduous or permanent teeth, it will compromise memory consolidation and learning (Lima et al. 2018).

### 3.2 Temporomandibular Disorders

TMD is a group of disorders affecting the temporomandibular joint (TMJ) or muscles, leading to pain and dysfunction in TMJ and surrounding structures (Sharma et al. 2011; Lomas et al. 2018). In addition, clicking, popping, or crepitus in TMJ, as well as difficulty in mouth opening, is frequently encountered in these diseases (Lomas et al. 2018). In adults, around 28% of patients with TMD present comorbidity of OSA (Smith et al. 2009), and OSA patients also present TMD pain more frequently than healthy controls (LeResche 1997). The prevalence of TMD in children and adolescents varies, ranging from 7.3% to 30.4% in children and adolescents at 10–19 years of age (Christidis et al. 2019), in which 1%–2% of children and 5% of adolescents require urgent treatment due to the severity of the disease (Athanasiou 2003). The intensity of pain from TMD has been linked to poor sleep quality in adolescents (Fernandes et al. 2022). In addition, adolescent patients with TMD‐derived pain often present symptoms of insomnia (Palermo et al. 2011), which is associated with impaired sleep latency and quality and subsequently developed depressive symptoms (Fernandes et al. 2022). Adult TMD patients with pain may have comorbidities other than insomnia (e.g., sleep apnea) (Smith et al. 2009).

### 3.3 Dentofacial Deformities

It has been widely acknowledged that maxillary and mandibular abnormalities are associated with morphological changes in the upper airway (Nargozian 2004). Adenoid hypertrophy often induces narrowing of the upper airway and aggravation of malocclusion in children (Wang, Qiao, et al. 2021). Moreover, the adenoid face is often accompanied by labial incompetence and an increase in mandibular plane angle (Heit et al. 2022). These conditions may significantly lower the sleep quality in these young patients. In contrast to adults, adenoid hypertrophy is considered one of the major anatomical causes for OSA in children (Venekamp et al. 2015), frequently leading to open‐mouth breathing (Durgut and Dikici 2019), and accompanied by longer sleep latency and worse sleep quality (Wang, Qiao, et al. 2021). A dentist is able to identify the maxillary deficiency or mandibular discrepancy to the cranial base.

## 4 Obstructive Sleep Apnea Affects Oral Health

OSA is a sleep disorder characterized by collapse of the pharynx during sleep (Jimenez et al. 2017), which affects up to 5.7% of children (Marcus et al. 2012). OSA may affect oral health by contributing to the alterations of maxillofacial structures, as evidenced by multiple studies showing that children with OSA may more frequently present crossbite (Ikavalko et al. 2012), narrow upper dental arch and shortened lower dental arch (Pirila‐Parkkinen et al. 2009), altered occlusions (Castilho et al. 2020), hypertrophy of tonsils and posterior‐rotation of the mandible (Heit et al. 2022), elongated soft palate, high arched palate or increased tongue size (Ngiam and Cistulli 2015). In clinical practice, dentists may identify a series of signs for OSA, such as redness of the soft palate and uvula area, narrow palate, enlarged tongue, torus mandibularis and retrusive jaw, together with dry mouth and bruxism (Berggren et al. 2022), so dentists are in a good position identifying these signs, their possible reasons, as well prevention of its unfavorable outcomes. These clinical observations all indicate a strong correlation between OSA and dental issues.

### 4.1 OSA and Periodontitis

In children with an average age of 12.3 years, dental caries and periodontitis are more frequently present in children with OSA compared with those without OSA (Tamasas et al. 2019). In addition, in children aged 8–17 years, higher scores for decay‐missing‐filled (DMF) for permanent teeth, pocket probing depth (PPD), and bleeding on probing (BOP) are more often present in children with SDB compared to those without SDB (Grillo et al. 2019). This correlation is also confirmed in children with Down syndrome, with levels of the gingival index and bleeding on probing significantly higher in children with OSA than those without OSA (Durhan et al. 2019). The presence of OSA affects children's quality of life, which is indicated by a higher score for the Child Oral Health Impact Profile (COHIP) and a lower score for the oral health and oral health‐related quality of life (OHRQoL) (Tamasas et al. 2019).

### 4.2 Osa and Mouth Breathing

Breathing through the nose is considered as the healthiest and most effective way to breathe (Tamkin 2020). Breathing through the mouth induces specific orofacial features, including incompetence of the upper lip, retro‐positioned hyoid bone, narrow dental arch, as well as other features, known as the “adenoid face” (Raffat and ul Hamid 2009; Koca et al. 2016). Various studies have reported the association between mouth breathing and craniofacial changes in pediatric patients (Kluemper et al. 1995). For instance, children with mouth breathing frequently present mandibular retrusion (El Aouame et al. 2016), backward and downward rotation of the mandible, increased mandible plane angle (Harari et al. 2010), narrow palate, severe crowding of the maxilla (Huynh et al. 2011), higher prevalence of anterior and posterior crossbite (Jimenez et al. 2017), as well as higher vertical dimension in the lower anterior face (Chambi‐Rocha et al. 2018). These morphometric changes not only present as features of mouth breathing but also as risk factors for mouth breathing. Specifically, a short and retruded mandible is considered a risk factor for mouth breathing in children at 5–12 years of age, and lower anterior height is considered a risk factor for mouth breathing in adolescents at 13–18 years of age with Class II malocclusion (Rossi et al. 2015). Compared to nasal breathing, children with mouth breathing present malocclusion more frequently (D'Ascanio et al. 2010). In children at 8–11 years of age, mouth breathing has been associated with risk for Class II dental malocclusion (Lee et al. 2021). Malocclusion is widely detected in children and adolescents, affecting sleep and school performance. It has been revealed that malocclusion is present in 40.4% of the pediatric population at 1–19 years of age, 40.9% of which showed significant improvement in sleep and learning following orthodontic therapy (Saccomanno et al. 2022).

Importantly, children with mouth breathing also have lower oxygen saturation compared with nasal‐breathing children (Jimenez et al. 2017); therefore, mouth breathing at night, together with snoring, has been considered a risk factor for OSA (Heit et al. 2022; Paduano et al. 2019). Furthermore, the craniofacial changes (e.g., mandibular retrusion with posterior rotation, steep mandibular plane) frequently present in mouth breathing are linked to increased risk of SDB in children (Flores‐Mir et al. 2013). For instance, mouth breathing is associated with SDB developed later in children at 6–11 years of age (Ikavalko et al. 2018). Specifically, it is present in about 89% of children with OSA, who also frequently encounter poor oral health (Kawashima et al. 1999). As features of OSA, mouth breathing as well as apnea hypopnea index (AHI) have been linked to risk for periodontitis (Seo et al. 2013).

## 5 Other Sleep Disturbances Affects Oral Health

### 5.1 Sleep Bruxism

Bruxism, which is characterized by repetitive clenching or grinding of teeth with or without bracing of the mandible, may occur during daytime and nighttime (Lobbezoo et al. 2013). Previous systematic review has shown that sleep bruxism was present in 3.5%–49.6% of children and adolescents (Manfredini et al. 2013). Sleep bruxism is associated with mouth breathing, snoring, difficulties initiating sleep, disturbed sleep, as well as short sleep duration (Guo et al. 2017). In participants aged ≥ 15 years, OSA increases the risk for sleep bruxism (Ohayon et al. 2001). Utilization of mandibular advancement devices has been proposed for sleep bruxism and sleep‐related breathing disorders. In fact, secondary sleep bruxism and comorbid sleep disorders are frequently encountered in clinical practice; dentists may be aware of the disease and refer the patients to a specialized clinic.

### 5.2 Disturbances In Sleep Profile

Approximately 31% of children at 6–13 years of age have difficulties in initiating and maintaining sleep (Spruyt et al. 2005). The disturbances in sleep profile not only affect children's general health and healthy thrive, but may also specifically affect dental health.

First, disturbance in sleep duration may participate in the development of oral problems. Gingival pain and bleeding, as well as pain in other intra‐oral regions, are more frequently present in individuals with shorter sleep duration compared with those with longer sleep duration (Choi et al. 2021). Similar findings have been revealed in children. Even in children at 3 years of age, short sleep duration is associated with increased risk of dental caries (Chen et al. 2018), and sleep duration less than 8 h poses a 30% increase in risk of dental caries (Sardana et al. 2023).

Second, optimized bedtime is not only crucial for healthy thriving but also for reduced risk of dental problems. Late bedtime is associated with an increased number of dental caries in both children and adolescents (Chen et al. 2018; Nishide et al. 2019). The incidence of dental caries is 66%–71% higher in children with interrupted bedtime (Sardana et al. 2023). The incidence of dental caries increases by 20% with every hour of bedtime delay after 8 pm in children at 10 years of age (Alqaderi et al. 2020), and if they go to bed after 11 pm, the incidence of developing dental caries will be 74%–85% higher (Sardana et al. 2023). Similarly, in a cross‐sectional study in adolescents at 12–18 years of age, individuals with late bedtime present a higher risk for toothache than those who sleep earlier (Choi et al. 2021).

Thirdly, disturbance in sleep quality also contributes to the development of dental caries and toothache. In addition, previous studies have shown that children with low quality of sleep is at higher risk of toothache (Choi et al. 2021), suggesting a potential link between sleep quality and dental health.

Finally, certain chronotypes may also be potentially correlated with dental caries. A previous study on adolescents aged 15 and 16 years old revealed a significantly higher risk of dental caries in individuals of evening type compared with those with other chronotypes (Lundgren et al. 2016). This may be due to a potential change in circadian rhythm or be correlated with lifestyle at night.

## 6 Mechanisms

Several mechanisms may underlie the pathophysiology of the bidirectional role between dental diseases and sleep disturbances.

### 6.1 Alteration of Oral Microorganism Colonization

Oral microbiota refers to microorganisms residing in the oral cavity (Dewhirst et al. 2010). It has been suggested that an imbalance of oral microbiota contributes to dental caries and periodontitis (Philip et al. 2018; Costalonga and Herzberg 2014), as well as to various other dental and systemic diseases (Saikaly et al. 2018; Reddy et al. 2018; Roszyk and Puszczewicz 2017). Mouth breathing may affect self‐cleaning of the oral cavity, which may subsequently induce gingivitis and an increase in oral bacterial colonization (Kinane 2001; Cronin et al. 2008), and progressively lead to progression of periodontitis (Carra et al. 2016). 16S rRNA analysis revealed that the relative abundance of *Firmicutes*, *Proteobacteria*, *Bacteroidetes*, *Fusobacteria,* and *Actinobacteria* in oral microbiome composition, as well as five metabolites of digestive‐tract‐related microbiota in urine samples, were different between OSA and control groups of children at 3–11 years of age (Xu et al. 2018). This could be due to altered oxygen concentrations under intermittent hypoxia of OSA, which could markedly affect the gut microbiome (Moreno‐Indias et al. 2015). Since the oral cavity is part of the digestive tract, OSA could then lead to oral dysbiosis. Therefore, the alteration of colonization of oral microorganisms may explain in part why children with OSA may be more vulnerable to dental caries and periodontitis. The altered metabolites may be explained by adenoid hypertrophy, which is an important and common issue in pediatric OSA patients, and is involved in diverse metabolic pathways (Kheirandish‐Gozal et al. 2013). In addition, late bedtime and short sleep duration in association with pediatric overweight/obesity have been linked with increased levels of mutans *Streptococci* (Arvidsson et al. 2016), which could result from night eating habits (Pieper et al. 2012). Night eating may be partially explained by a lower level of leptin and a higher level of ghrelin detected in the saliva of children with late bedtime, which are linked with a higher incidence of dental caries (Alqaderi et al. 2020). These clinical results have provided evidence that sleep disturbances may result in oral diseases in children by affecting oral microorganism colonization. As a matter of fact, *Streptococcus* and *Actinomyces* were found significantly increased in pediatric patients with severe dental caries (Jiang et al. 2013).

On the other hand, the composition of the oral microbiome shifts during sleep, especially seen in children with dental decay, as the number of decay‐causing bacteria rapidly increases in saliva during sleep (Sotozono et al. 2021), potentially leading to systematic inflammation (Cardoso et al. 2018) and further sleep disruption.

### 6.2 Impairment of Immune Defense and Persistent Inflammation

Saliva plays a crucial role in lubrication, immune defense, as well as other functions. Salivary flow follows a circadian rhythm – the secretion is low in the morning, and gradually increases in the afternoon until evening, then reduces subsequently (Furukawa et al. 2005). Therefore, disturbance of the rhythm affects the secretion of saliva, which subsequently affects oral hygiene and causes dental caries. In addition, changes in components of saliva may also contribute to the pathogenesis of these diseases. An animal study has previously shown that total sleep deprivation is associated with decreased salivary flow rate, reduced salivary secretion of IgA, and elevated salivary amylase activity, which have been shown as risk markers for dental caries and decay and stress‐induced activity of the sympathetic nervous system, respectively (Lasisi et al. 2017). In shift and night workers, short sleep duration is associated with increased production of the pro‐inflammatory cytokine interleukin‐6 (IL‐6) in saliva, indicating the potential of using salivary IL‐6 instead of plasma IL‐6 as a robust marker for future sleep deprivation studies (Reinhardt et al. 2016). A higher salivary IL‐6 level has been observed in patients with periodontitis compared to healthy controls (Isola et al. 2021).

Inflammation is a mutual factor underlying the pathogenesis of both OSA and periodontitis (Al‐Jewair et al. 2015). Systemic inflammation is an overlapping feature of both periodontitis and OSA (Khodadadi et al. 2022; Nadeem et al. 2013). In OSA, a higher level of inflammation is accompanied by a higher prevalence of dental plaque and other periodontal parameters (Gamsiz‐Isik et al. 2017). Dental plaque is a leading cause of gingivitis and periodontitis in the absence of oral hygiene (Patil et al. 2014), and the frequency of oral hygiene is negatively correlated with gingival inflammation (Kolawole et al. 2011). Additionally, Intermittent hypoxia presented in OSA leads to oxidative stress and inflammation, both of which contribute to damage of the periodontal tissue (Lavie 2015; Oishi et al. 2016).

On the other hand, chronic inflammation caused by untreated or uncontrolled dental issues like gum diseases can negatively impact sleep patterns and sleep quality by increasing the release of pro‐inflammatory cytokines such as IL‐6 and tumor necrosis factor‐ɑ (TNF‐ɑ), which may subsequently lead to altered immune responses (Movahed et al. 2023).

### 6.3 Chronic Pain

All orofacial pain may disturb a patient's sleep, leading to unrefreshed sleep and affecting performance and mood the next day. Pain during sleep could potentially induce full awakening (Marshansky et al. 2018). One widely studied underlying pathway that links chronic pain and sleep is the inflammatory pathway, based on observations from a wide range of clinical studies. For instance, in comparison to healthy controls, female patients with TMD who had worse sleep quality and insomnia presented upregulated plasma cytokine levels of IL‐6, IL‐1β, and TNF‐ɑ (Park and Chung 2016; Hunt et al. 2022; Lerman et al. 2022). Levels of these cytokines vary with the sleep‐wake cycle, and may interfere with REM or NREM sleep in a dose‐ and time‐dependent manner (Krueger et al. 2007). Furthermore, as a neurotransmitter involved in pain modulation, serotonin (also known as 5‐HT) was found to decrease in the frontal cortex and hippocampus in a REM sleep deprivation animal model (Lautenbacher et al. 2006).

Conversely, poor sleep quality may also result in aggravation of orofacial pain. Sleep deprivation may increase activity in the cortex and reduce activity in the striatum, insula, and nucleus accumbens, and subsequently modify structures related to pain processing in the brain (Krause et al. 2019; Seminowicz et al. 2019). In addition, the effect of sleep restriction on nociception could be regulated by interactions across multiple GABAergic signaling pathways and reduced monoaminergic activity in the nucleus accumbens (Sardi et al. 2024).

## 7 Treatments

As reported, tooth brushing with proper techniques, such as employing the modified Bass technique, is effective in reducing dental plaque in children aged 6–8 years (Patil et al. 2014). It has been reported in preschool children (aged 3–6 years) with dental caries that dental treatment eliminating pain and inflammation could significantly improve sleep disturbances (measured by Child Sleep Habits Questionnaire [CSHQ]) along with growth parameters and quality of life, suggesting the importance of an early dental intervention for caries (Gunay et al. 2023).

Traditionally, functional oral appliances are used to correct Class II malocclusion due to mandibular retrusion in pediatric patients. With these appliances, the mandibular plane and lower facial height will increase, while the overbite and overjet will decrease (Almeida et al. 2006; Pliska et al. 2014). The commercially available fixed functional appliances (Herbst and AdvanSync) both significantly increased the airway dimensions and tongue parameters in teenage patients with skeletal Class II malocclusion after an 8‐month appliance therapy, indicating an extended use of these appliances in OSA (Arora et al. 2024). When comparing the effect of a Twin block (with correction of the mandibular retrognathism) and a fixed appliance (without correction of jaw relationship) on the improvement of SDB related parameters in orthopedic patients (at an average age of 10 years) with skeletal Class II Division 1 malocclusion, the Twin block group showed a significantly larger improvement in oropharyngeal airway volume and nocturnal breathing (Radwan et al. 2024). For pediatric patients (aged 3–9 years) with tonsillar hypertrophy and chronic tonsillitis, the subtotal intracapsular tonsillectomy significantly improved sleep disorders of these young patients and was suggested to be the first choice (Wang, Dang, et al. 2021). Nonsurgical treatment for pediatric OSA includes rapid maxillary expansion (RME) (Gomes et al. 2014), mandibular advancement, and myofunctional therapy. RME is an appliance that opens the midpalatal suture to expand the maxillary arch, correct posterior crossbites, and improve facial symmetry (Stark et al. 2018). It is especially effective in younger patients with a high, narrow palate and crossbite (Gozal et al. 2020). Previous studies have highlighted the effectiveness of RME (Huynh et al. 2016). Myofunctional therapy includes a series of strengthening exercises aiming to improve muscle tone as well as muscle function.

As per sleep bruxism, it was reported in two randomized controlled trials (RCTs) that both low‐level laser sessions and physical therapy of relaxation seemed to be promising alternative therapeutic options for children (6–12 years) with a history of sleep bruxism (Rashed et al. 2025; Salgueiro et al. 2021). The relaxation therapy (administered in the form of mindfulness meditation via relaxation audio) also demonstrated effectiveness when combined with sleep hygiene measures in the management of probable sleep bruxism in children (3–8 years) (Amaral et al. 2025). In another RCT observing the effect of a lifestyle‐related interventional program called “Food, Fun and Family (FFF)” on children aged between 4 and 8 years with high‐frequency parental‐reported sleep bruxism, participants in experimental group received a 12‐week food and screen parenting intervention and achieved a more significant reduction in consumption of added sugar and screen time, leading to decreased sleep bruxism frequency (Restrepo‐Serna et al. 2025).

## 8 Conclusions and further Recommendations

Untreated dental diseases may lead to disturbances in daily activities such as sleeping and eating. Therefore, upon identification of dental caries, gingivitis, periodontitis, as well as other dental problems, a follow‐up question on a child's quality of life, including sleep, is important, as this may subsequently affect a child's sleep initiation and maintenance, as well as sleep duration and sleep quality. Management of these issues as a whole will facilitate the prevention of further decrease in cognitive and psychological functions.

Multiple studies have revealed a strong correlation and bidirectional relationship between dental diseases and sleep disturbances. Moreover, in clinical practice, there is a high occurrence of sleep disturbances, especially SDB and OSA, among pediatric patients who come to seek orthodontic treatment. In pediatric patients, many oromorphological changes and changes that occur following sleep disturbances are modifiable following proper intervention. Early identification and realization of dental diseases are important for the initiation of intervention to prevent the development and progression of sleep disorders and subsequent negative outcomes. Hence, dentists and orthodontists are in a critical position to identify signs and symptoms that require further evaluation for a solid diagnosis of sleep disturbances. In addition to the identification of orofacial features, the disturbed behavior and mood of a pediatric patient at the clinic, together with a history of his/her poor school performance, may also flag a cohort that needs further investigation. More importantly, dentists could better understand the specific need of patients, their psychological and cognitive status, and assist with decision‐making.

There were several limitations for the studies reviewed, where optimized studies are needed to validate these findings and provide information for future directions. First, cross‐sectional studies cannot prove direct causality, and therefore, well‐planned longitudinal studies, especially long‐term longitudinal studies, are needed to suggest causal links among these parameters. Second, various measurements were subjective, and many were questionnaires that were responded by parents. Therefore, questionnaires should be carefully selected, and objective measurements such as actigraphy and polysomnography are desired in future studies. Third, potential covariates were not sufficiently addressed by some of the studies, and potential confounding factors such as age group, ethnicity, and socioeconomic status should be explored in studies in the future. In addition, sample size should also be considered for future designs.

## Author Contributions

Conceptualization: Minjun Dong. Investigation: Yaqiong Zhang, Wenqi Yang, and Qi Sun. Data curation: Yaqiong Zhang, Wenqi Yang, and Qi Sun. Writing – original draft: Yaqiong Zhang. Writing – review and editing: Wenqi Yang, Qi Sun, and Minjun Dong. Visualization: Yaqiong Zhang. Funding acquisition: Yaqiong Zhang. Supervision: Minjun Dong.

## Ethics Statement

There are no human participants in this article and informed consent is not applicable.

## Conflicts of Interest

The authors declare no conflicts of interest.

## Data Availability

Data sharing is not applicable to this article as no new data were created or analyzed in this study.
